# Prevalence and associated factors of selling sex among men who have sex with men (MSM) in Latin America: results from the Latin American MSM Internet Survey in 18 countries (LAMIS-2018)

**DOI:** 10.1136/bmjgh-2025-021058

**Published:** 2025-12-19

**Authors:** Mariano Salazar, Nicolas Lorente, Axel Jeremias Schmidt, Kai Jonas, Signe Svallfors, Anna Mia Ekström, Torsten Berglund, Carlos F Cáceres, Susanne Strömdahl, Valeria Stuardo, Jordi Casabona

**Affiliations:** 1 Department of Global Public Health, Karolinska Institutet, Stockholm, Stockholm County, Sweden; 2 Community-Based Research Laboratory, Coalition PLUS, Pantin, Île-de-France, France; 3 CEEISCAT, Barcelona, Spain; 4 Sigma Research, Department of Public Health, Environments and Society, London School of Hygiene & Tropical Medicine, London, England, UK; 5 Faculty of Psychology and Neuroscience, Maastricht University, Maastricht, Netherlands; 6 Department of Sociology, Stockholm University, Stockholm, Stockholm County, Sweden; 7 Department of Infectious Diseases (Venhälsan), South General Hospital, Stockholm, Sweden; 8 Universidad Peruana Cayetano Heredia, Lima District, Lima Region, Peru; 9 Department of Global Public Health, Karolinska Institute, Stockholm, Stockholm County, Sweden; 10 Public Health Institute, Universidad Andrés Bello, Santiago, Santiago Metropolitan Region, Chile; 11 Global Health Program, School of Public Health, University of Chile, Santiago, Santiago Metropolitan Region, Chile; 12 Germans Trias i Pujol Research Institute (IGTP), Barcelona, Spain

**Keywords:** Global Health, Cross-sectional survey

## Abstract

**Introduction:**

Selling sex has been associated with negative social and health outcomes, but most studies have been limited geographically and have not distinguished between selling and buying sex. This study assesses prevalence and factors associated with selling sex in the last 12 months among men who have sex with men (MSM) in 18 Latin American countries.

**Methods:**

Data were collected in 2018 through the Latin American MSM Internet Survey, a cross-sectional online survey. Of 64 655 participants, 9585 were excluded due to data inconsistencies on age and partner status, and 1728 due to missing outcome data, yielding an analytic sample of 53 342. Multivariable logistic regression was used for analysis.

**Results:**

Overall, 6.9% (10.3% among MSM aged 18–24) reported selling sex in the previous year. Higher odds of selling sex were associated with younger age, low education, being born abroad, low financial coping, substance use, potential alcohol dependency, early sexual debut with a male partner, low sexual agency and sex with women. High educational level and having a steady male partner were associated with lower odds.

**Conclusions:**

Key factors associated with selling sex among MSM in Latin America include socioeconomic, behavioural and relational variables. Harm reduction and preventive interventions may be particularly needed among younger MSM. Codeveloping these interventions with the MSM community can ensure sustainability, relevance and strengthen providers’ ability to offer individualised, respectful care. Longitudinal and qualitative studies are needed to monitor long-term health and tailor interventions to individual needs.

**WHAT IS ALREADY KNOWN ON THIS TOPIC?**

- The prevalence of selling sex among men who have sex with men (MSM) has been studied in Latin America (LatAm), but findings are often limited to specific countries or subnational regions.
- Prior research in LatAm on transactional sex among MSM frequently employed definitions that do not distinguish between selling and buying sex.
- Selling sex among MSM in LatAm has been associated with certain sociodemographic factors (eg, poverty, lower educational attainment), HIV-positive status and a history of sexually transmitted infections.

**WHAT THIS STUDY ADDS?**

- Latin America MSM Internet Survey-2018 is the largest survey to date of MSM in LatAm, with over 53 000 participants from 18 countries across the region.
- Our analysis found that the prevalence of selling sex was significantly higher among younger MSM, with approximately 1 in 10 MSM aged 18–24 reporting having sold sex at least once in the past 12 months.
- The study provides a comprehensive assessment of substance use among MSM in LatAm, showing that the use of specific substances (stimulants, gamma hydroxybutyrate/gamma butyrolactone, ketamine and heroin), alongside factors such as being born abroad, low sexual agency, younger age at first sex with a male and having sex with women, are associated with increased odds of selling sex in the past 12 months.

**HOW THIS STUDY MIGHT AFFECT RESEARCH, PRACTICE OR POLICY?**

- This study underscores the need for health systems in LatAm to integrate structural, behavioural and relational factors into strategies addressing selling sex among MSM.
- Public health services must enhance provider training to recognise and respond to the specific needs of MSM engaged in selling sex, ensuring care that is individualised, stigma-free and rights-based.
- The findings highlight the need for regional policies that promote social inclusion, protect the rights of MSM who sell sex and ensure equitable healthcare access—especially for younger MSM—while emphasising the importance of longitudinal and qualitative research to guide evidence-based policies, monitor health outcomes and design responsive interventions.

## Introduction

 Selling sex, defined as receiving money, services or goods in exchange for sex, has been associated with a higher likelihood of a variety of poor health outcomes due to underlying socioeconomic factors that may negatively impact social and health outcomes.^1^,^6^ Among men who have sex with men (MSM), however, this is less well researched than among women having sex with men, but studies show a higher likelihood of being diagnosed with HIV^2^,^6^ or other sexually transmitted infections (STI),^4^,^6^ experiencing violence^5 7 8^ and higher substance use or hazardous alcohol consumption.^3^,^57^ Other variables such as being young,^3 5^ unemployed or having a limited income,^3 5^ low education,^3 5^ being transgender^7^ and having had an early sexual debut^6^ have been identified as factors associated with selling sex among MSM, though this evidence comes mostly from cross-sectional studies that cannot attribute causally to selling sex.

The prevalence of selling sex varies based on the time frame used to assess it, the subpopulation studied and the definitions applied. Multicountry studies in Europe^4^ and Latin America^5^ using convenience sampling found that 4.5%–7.5% of MSM had sold sex in the previous 12 months while a study using response-driven sampling in nine Latin American cities (in Guatemala, El Salvador, Nicaragua, Costa Rica and Panama) found a much higher prevalence of selling sex in the last 30 days, ranging from 12% to 40%.^5^,^79 10^ Prior studies in Latin America on transactional sex among MSM,^5^,^79 10^ often used a definition of transactional sex that does not differentiate between selling and buying,^9 11^ or are geographically limited to one country^6 9^ or region.^7^ This study addresses this knowledge gap by assessing the prevalence and associated factors of selling sex among MSM in 18 countries in Latin America using the Latin America MSM Internet Survey (LAMIS), the largest survey on MSM, with the overall aim to strengthen and better target healthcare services and harm reduction programmes^12^ for MSM in Latin America.

## Methods

### Study design and sampling

We used data collected through LAMIS, a cross-sectional online survey conducted simultaneously in 18 Latin American countries (Argentina, Bolivia, Brazil, Chile, Colombia, Costa Rica, Ecuador, El Salvador, Guatemala, Honduras, Mexico, Nicaragua, Panama, Paraguay, Peru, Suriname, Uruguay and Venezuela) from 24 January to 13 May 2018.^13^ The study primarily recruited participants through online dating apps such as Grindr, ROMEO and Hornet (75%) and through social networks via national community-based organisations.^13^

The survey was designed and implemented through a collaboration between The Ibero-American Network of Studies on Gay Men, other MSM and Transgender People (Right PLUS), researchers from Germany and the Netherlands and Sigma Research (London School of Hygiene & Tropical Medicine). A detailed description of the study design can be found elsewhere.^13^ No sample size was calculated a priori, as the aim was to reach as many MSM as possible in each country and collect as much data as possible. This approach also aimed to provide respondents with deeper self-insight and knowledge about HIV/STI testing and sexual behaviour. Brazil and Mexico were the biggest contributors, with 18 139 and 14 957 participants, respectively.^13^

### Questionnaire

The Latin American questionnaire was adapted from the European MSM Internet Survey (EMIS-2017), a multicountry study collecting a wide range of epidemiological and behavioural data on MSM,^14^ but piloted to ensure clarity and cultural relevance.^13^ The data were collected in Spanish, Portuguese and Dutch.

The outcome variable—selling sex at least once in the previous 12 months—was assessed with the question: *When was the last time you were paid by a man to have sex with him? By paid, we mean he gave you money, gifts or favours in return’*. Those who indicated having been paid for sex were asked how many times they had sold sex during this period, with the following response options: (a) *1–2 times, (b) 3–10 times, (c) 11–50 times and (d) more than 50 times. *

A preliminary analysis of the data showed that very few participants had sold sex 11 times or more. Therefore, these categories were combined with those who reported selling sex 3–10 times. The resulting variable included three categories: (1) *No selling in the previous 12 months*, (2) *1–2 times* and (3) *3 or more times*. A multinomial logistic regression analysis revealed that the variables significantly associated with selling sex *1–2 times* and *3 or more times* were mostly the same, with comparable point estimates (see online supplemental file 1). To simplify the presentation of findings, the outcome variable was therefore dichotomised to indicate whether the respondent had sold sex at least once in the past 12 months or not.

We measured the following demographic variables: age in years (18–24, 25–29, 30–34, 35–39, 40–44, 45–49, 50–54 and 55 years or more), education (less than high school, high school, university, master’s degree or above) and current steady male partnership (yes, no, it is complicated). Participants’ financial coping was evaluated by asking which of the following statements best reflected their current income situation (living really comfortably, living comfortably, neither comfortable nor struggling, struggling or really struggling).

Participants’ country of residence included the following: Colombia, Peru, Mexico, Ecuador, Argentina, Brazil, Chile, Venezuela, Bolivia, Paraguay, Uruguay, Suriname, Panama, Costa Rica, Nicaragua, Honduras, Guatemala and El Salvador. Participants were also asked whether they were born in their country of residence or abroad. A preliminary analysis of the data showed that a significant proportion of those born abroad were Venezuelans; thus, in order to better describe the data, we divided those born abroad into three categories: (1) born abroad in Venezuela, (2) born abroad in another LAMIS country and (3) born abroad in other countries.

Substance use in the previous 12 months was measured by asking participants if they had used several substances, which were later categorised as follows: (1) any stimulant substance use (ecstasy, amphetamines, methamphetamine, mephedrone, cocaine and stimulants other than mephedrone), (2) heroin use, (3) ketamine use, (4) cannabis use and (5) gamma hydroxybutyrate (GHB) or gamma butyrolactone (GBL) use.

Potential alcohol dependency was measured using the CAGE-4 (Cut, Annoyed, Guilty and Eye-opener) screening questionnaire.^15^ It consists of four yes/no questions asking the following: *Have you tried to cut down on your drinking?, Have people annoyed you by criticising your drinking?*, *Have you felt bad or guilty about your drinking?* and *Have you taken a drink first thing in the morning to steady your nerves or get rid of a hangover?*. According to previous research, answering positively to two or more questions is considered potential alcohol dependency and was scored as such in this paper.^15^

Sexual agency was measured by whether the participants agreed with two statements: (1) *The sex I have is always as safe as I want it to be* and (2) *I find it easy to say no to sex I don’t want*. Originally measured using a 5-point Likert scale, we dichotomised these indicators into *yes* (agree, strongly agree) and *no* (disagree, strongly disagree, not sure). We also included two variables capturing sexual behaviours: (1) Whether the participants had had sex with a woman in the previous 12 months or more than 12 months ago (yes/no) and the participants’ age at first sex with a man (*don’t know*, ≤13 years, 14–17 years and ≥18 years).

### Analysis

From the original dataset of 64 655 participants, 9585 participants were excluded due to inconsistencies regarding age and partner status, and an additional 1728 participants who did not have data on the outcome variable. Thus, our analytic sample contained 53 342 participants. We compared characteristics of included and excluded participants (online supplemental file 2) and found only small differences (generally 1–2 percentage points, all <5%). Given the large sample size, these differences are unlikely to have meaningfully affected regression estimates.

The data were very complete, since 94% (51 617) of the sample had complete data on all variables included in the multivariable model and most variables had less than 1% missing values (except age at first sex with a male with 3.4% of the information of this variable missing (1852/53 218). We, therefore, used a complete case analysis for the multivariable model.

We first conducted univariate and bivariate analyses. Frequencies and percentages were used to describe the data. χ^2^ tests were used to assess statistical differences between groups (eg, percentage selling sex by age categories). Logistic regression was used to obtain unadjusted and adjusted ORs (AOR) and 95% CIs for the association between selling sex at least once in the previous 12 months (main outcome) and demographic, substance use and sexual behaviour variables. Variables that were significantly associated with selling sex in previous studies were included in the multivariable model (age, education, country of residence, country of birth, current partner status, financial coping, potential alcohol dependency, substance use, sex with women, sexual agency and age at first sex with a man).^4^,^616^ All the variables above were also significant in the bivariate analyses (p<0.05).

Although the data were hierarchical, multilevel modelling was not feasible given the small number of higher level units (18 countries; stable estimation typically requires ≥30 clusters).^19^ To address within-country dependence, we included country fixed effects, absorbing all time-invariant differences across the 18 countries.^19^ To enhance interpretability, AORs were converted into average adjusted probabilities, using the margins command in Stata V.17. These estimates inherently account for the baseline prevalence and the confounding effects of all model covariates. Multicollinearity was evaluated using tolerance and variance inflation factor (VIF) postestimation diagnostic tests. The overall model VIF was 1.13, with individual variable VIFs ranging from 1.00 to 1.52. No multicollinearity was detected. Multiplicative interaction was assessed between age, substance use and potential alcohol dependency.

### Patient and public involvement

The current survey was adapted from EMIS-2017, which was designed in collaboration with more than 200 academics, civil and government organisations from 50 countries.^14^ In LatAm, the survey was discussed and validated with non-govermental organizations (NGOs) and academics who are part of the Right PLUS network. In addition, local NGOs in the participating countries helped promote the study through their websites and other outreach activities. All collaborating organisations could have access to the database of their country, this allowed them to analyse their own data and disseminate them at national level.

### Ethical considerations

The informed consent form outlined the study’s objectives, identified the consortium of institutions responsible for data collection and indicated that participation was entirely voluntary and strictly anonymous, with no personally identifiable information collected. It also included the researchers’ contact information, described the potential risks—or lack thereof—associated with participation, and stated that all data would be stored on a secure, access-restricted server to ensure confidentiality. No financial incentives were provided to participants.

## Results

**Figure 1:**
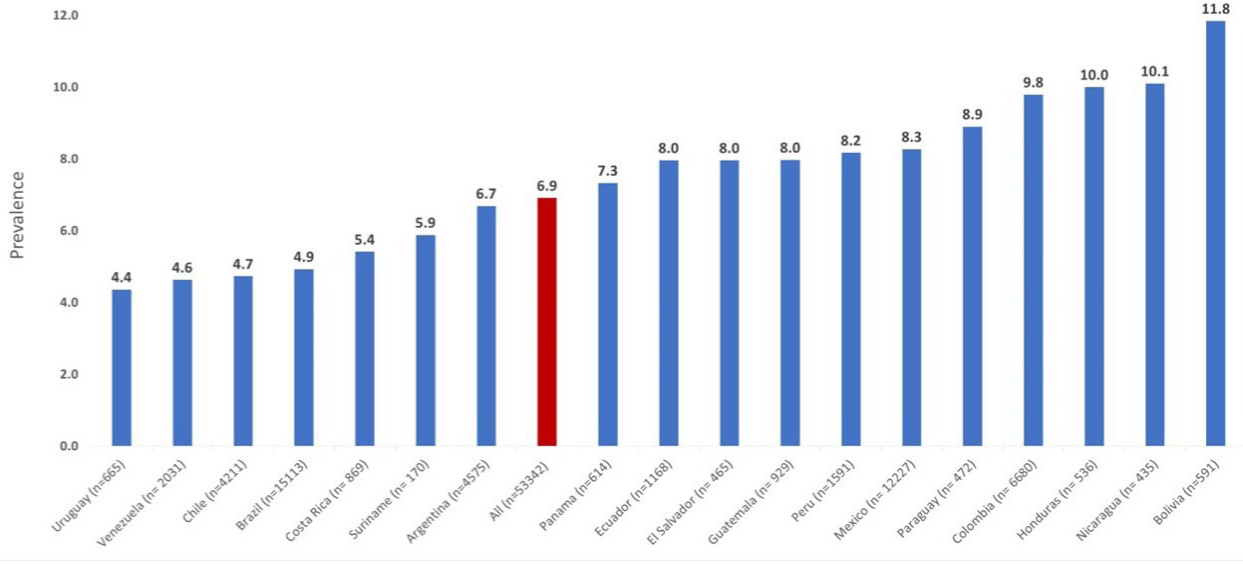
Prevalence of selling sex in the previous 12 months stratified by country (n=53 342).

**Figure 2:**
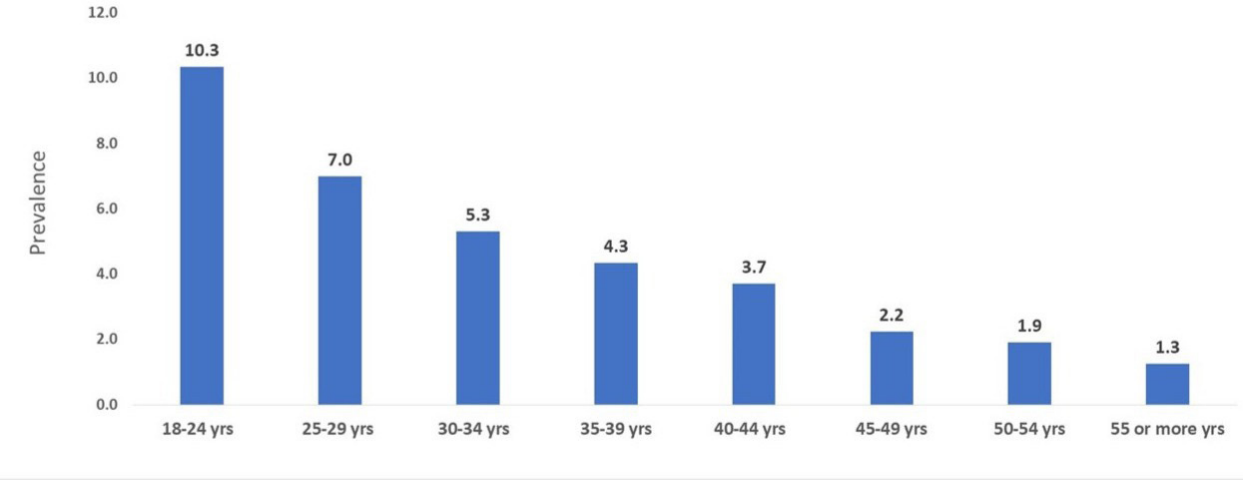
Prevalence of selling sex in the previous 12 months stratified by age (n=53 342).

The overall prevalence of selling sex at least once in the previous 12 months was 6.9% (95% CI 6.7 to 7.1). Of those, 4.1% (n=2193) sold sex 1–2 times, 1.9% (n=1032) 3–10 times and 0.6% (n=315) 11–50 times and 0.27% (n=144) more than 50 times. The highest overall prevalence was found in Bolivia (11.8%) and the lowest in Uruguay (4.4%) (figure 1). Young MSM aged 18–24 years had the highest prevalence of selling sex (10.3%) while only 1.3% of those aged 55+ years had done so in the past year (figure 2).

Table 1 displays sample characteristics (table 1). Most respondents were young, as 33.4% were 19–24 years and 25.0% between 25–29 years. Many had a high educational level, 49.9% at university level and 40.3% reported that they were *neither comfortable nor struggling* with their current income when asked about financial coping (table 1). Most (95.6 %) were born in their country of residence (table 1). Of those living in a country but born abroad (n=2373), 41.6% (988/2373) were born in Venezuela, 40.9% (971/2373) were born in another LAMIS country and the rest elsewhere. We found that 42.8% (96/224) of those selling sex and born abroad were Venezuelans.

**Table 1:** Sample characteristics stratified by selling sex in the previous 12 months, column percentages shown

Characteristics	No		At least one time		Total	
	n	%	n	%	n	%
Age (years)*, n=**53 342**						
18–24	16 016	32.4	1848	50.2	17 864	33.4
25–29	12 384	24.9	930	25.2	13 314	25.0
30–34	8207	16.5	459	12.5	8666	16.3
35–39	5228	10.5	237	6.4	5465	10.3
40–44	3090	6.2	119	3.2	3209	6.0
45–49	2104	4.2	48	1.3	2152	4.0
50–54	1444	2.9	28	0.8	1472	2.7
55 or more	1185	2.4	15	0.4	1200	2.3
Education*, n=**53 241**						
High school or less	5078	10.2	558	15.2	5636	10.6
Technical education	10 202	20.6	1140	31.1	11 342	21.3
University	24 900	50.2	1670	45.5	26 570	49.9
Master or more	9392	19.0	301	8.2	9693	18.2
Country of birth*, n=**53 285**						
Born in country of residence	47 454	95.7	3458	93.9	50 912	95.6
Born abroad Venezuela	892	1.8	96	2.6	988	1.8
Born other LAMIS countries	877	1.7	94	2.5	971	1.8
Born non-LAMIS countries	380	0.8	34	1.0	414	0.8
Steady male partner*, n=**53 236**						
No/it’s complicated	36 312	73.3	3023	82.2	39 335	73.9
Yes	13 247	26.7	654	17.8	13 901	26.1
Financial coping*, n=**53 165**						
Living really comfortably	6847	13.8	409	11.2	7256	13.6
Living comfortably	14 116	28.5	753	20.6	14 869	28.0
Not comfortable/struggling	19 849	40.1	1576	43.0	21 425	40.3
Struggling	6462	13.1	638	17.4	7100	13.4
Really struggling	2227	4.5	288	7.8	2515	4.7
Sex with women*, n=**53 056**						
Never	31 030	62.8	2184	59.6	33 214	62.6
Yes, previous 12 months	4134	8.4	526	14.4	4660	8.8
Yes, more 12 months ago	14 231	28.8	951	26.0	15 182	28.6
Sex safe as planned *, n=**53 298**						
No	15 060	30.4	1373	37.3	16 433	30.8
Easy to say ‘No’ to sex *, n=**53 234**						
No	11 754	23.7	1101	29.9	12 855	24.2
Alcohol dependency*, n=**52 932**						
Yes	10 156	20.6	996	27.3	11 152	21.1
Age at first sex with a man*, n=53 051						
Don’t know	611	1.2	52	1.4	663	1.3
≤13 years	10 654	21.6	1130	30.8	11 784	22.1
14–17 years	17 478	35.4	1654	45.0	19 132	36.1
≥18 years	20 635	41.8	837	22.8	21 472	40.5
Stimulant use 12 months, n=53 012*†						
Yes	6890	13.9	929	25.4	7819	14.7
Heroin use 12 months, n=52 969*						
Yes	195	0.4	46	1.2	241	0.4
Ketamine use 12 months, n=52 969*						
Yes	1344	2.7	218	5.9	1562	2.9
Cannabis use 12 months, n=53 004*						
Yes	14 852	30.1	1379	37.7	16 231	30.6
GHB/GBL use 12 months, n=52 948*‡						
Yes	834	1.69	124	3.39	958	1.81

* χ2 test, p<0.05).

† Stimulant substances include cocaine, ecstasy, amphetamines, methamphetamine, mephedrone, synthetic stimulants other than mephedrone.

‡ Gamma hydroxybutyrate (GHB) and gamma butyrolactone (GBL).

LAMIS, Latin American MSM Internet Survey.

Regarding substance use in the last 12 months, the three most common substances were cannabis (30.6%), any stimulant substances (14.7%) and ketamine (2.9%). In addition, 21.1% of the participants were classified as potentially alcohol dependent (table 1). Most did not have a steady male partner or classified their male partnership as *complicated* (73.9%), and about two-thirds (62.6%) reported never having had sex with a woman (table 1).

When assessing sexual agency, almost one-third (30.8%) of respondents reported that they did not always have sex as safely as they wanted it to be, and almost one quarter (24.1%) reported that they could not easily say no to unwanted sex (table 1). As for respondents’ same-sex sexual debut, 22.1% were 13 years or younger, and 36.1% were 14–17 years old when they had their first sex with a male (table 1).

The comparison between crude and AORs reveals that many associations with selling sex in the past 12 months were influenced by confounding factors (table 2). In several cases (eg, age, heroin use, alcohol dependency, etc), the association weakened after adjustment, indicating that the crude estimates were inflated by other variables. Some associations, such as those for stimulant use and early sexual debut, remained strong, suggesting robust, independent effects. Overall, the adjusted analysis provides a clearer picture of which factors are truly associated with selling sex. In the following, we describe the adjusted estimates.

**Table 2:** Factors associated with selling sex at least once in the previous 12 months, unadjusted (OR), adjusted or (AOR), 95% CI, and average adjusted probabilities (APP) shown, n=51 530

Characteristics	OR (95% CI)	AOR (95% CI)	APP (95% CI)	% of change
Age (years)				
18–24	1.00	1.00	8.90 (8.4 to 9.32)	0.0
25–29	0.65 (0.59 to 0.70)	0.76 (0.69 to 0.83)	7.07 (6.63 to 7.51)	−20.6
30–34	0.48 (0.43 to 0.53)	0.60 (0.54 to 0.68)	5.78 (5.26 to 6.30)	−35.1
35–39	0.39 (0.34 to 0.45)	0.50 (0.43 to 0.58)	4.90 (4.28 to 5.51)	−44.9
40–44	0.33 (0.27 to 0.40)	0.43 (0.35 to 0.53)	4.27 (3.51 to 5.04)	−52.0
45–49	0.19 (0.14 to 0.26)	0.26 (0.19 to 0.36)	2.70 (1.92 to 3.47)	−69.7
50–54	0.16 (0.11 to 0.24)	0.22 (0.15 to 0.33)	2.33 (1.45 to 3.21)	−73.8
55 or more	0.10 (0.05 to 0.18)	0.17 (0.10 to 0.30)	1.85 (0.93 to 2.77)	−79.2
Country of residency				
Brazil	1.00	1.00	4.53 (4.20 to 4.86)	0.0
Argentina	1.38 (1.20 to 1.58)	1.50 (1.30 to 1.78)	6.56 (5.84 to 7.28)	+44.8
Chile	0.95 (0.81 to 1.12)	1.15 (0.97 to 1.36)	5.16 (4.46 to 5.86)	+13.9
Colombia	2.09 (1.87 to 2.33)	2.13 (1.89 to 2.41)	8.96 (8.30 to 9.62)	+97.8
Ecuador	1.66 (1.33 to 2.08)	1.69 (1.33 to 2.14)	7.31 (5.87 to 8.75)	+61.4
Mexico	1.73 (1.57 to 1.91)	2.30 (2.06 to 2.56)	9.54 (8.98 to 10.11)	+110.6
Peru	1.71 (1.41 to 2.08)	1.96 (1.60 to 2.41)	8.34 (6.99 to 9.69)	+84.1
Venezuela	0.93 (0.75 to 1.16)	1.12 (0.89 to 1.42)	5.09 (4.07 to 6.12)	+12.4
Bolivia	2.59 (1.99 to 3.36)	2.66 (2.01 to 3.51)	10.75 (8.38 to 13.13)	+137.3
Costa Rica	1.10 (0.81 to 1.49)	1.16 (0.84 to 1.61)	5.21 (3.72 to 6.70)	+15.0
El Salvador	1.66 (1.18 to 2.35)	1.80 (1.26 to 2.58)	7.72 (5.37 to 10.07)	+70.4
Guatemala	1.66 (1.30 to 2.14)	1.90 (1.46 to 2.46)	8.07 (6.34 to 9.79)	+78.1
Honduras	2.16 (1.61 to 2.88)	2.04 (1.50 to 2.79)	8.60 (6.38 to 10.83)	+89.8
Nicaragua	2.17 (1.57 to 2.98)	2.24 (1.59 to 3.14)	9.30 (6.70 to 11.90)	+105.3
Panama	1.52 (1.11 to 2.08)	1.84 (1.32 to 2.55)	7.84 (5.67 to 10.02)	+73.1
Paraguay	1.88 (1.36 to 2.60)	1.82 (1.27 to 2.60)	7.78 (5.44 to 10.12)	+71.7
Suriname	1.20 (0.63 to 2.29)	1.41 (0.67 to 2.95)	6.20 (2.11 to 10.29)	+36.9
Uruguay	0.87 (0.60 to 1.28)	0.96 (0.64 to 1.44)	4.40 (2.80 to 60.02)	−2.9
Country of birth				
Born country of residence	1.00	1.00	6.79 (6.57 to 7.01)	0.0
Born abroad Venezuela	1.47 (1.19 to 1.82)	1.48 (1.18 to 1.86)	9.59 (7.76 to 11.42)	+41.2
Born other LAMIS countries	1.47 (1.18 to 1.82)	1.48 (1.17 to 1.87)	9.56 (7.71 to 11.42)	+40.8
Born non-LAMIS countries	1.22 (0.86 to 1.74)	1.49 (1.03 to 2.71)	9.69 (6.64 to 12.74)	+42.7
Education				
High school or less	1.00	1.00	9.76 (8.95 to 10.57)	0.0
Technical education	1.01 (0.91 to 1.13)	0.82 (0.73 to 0.93)	8.24 (7.76 to 8.71)	−15.6
University	0.61 (0.55 to 0.67)	0.60 (0.54 to 0.67)	61.7 (5.89 to 6.46)	−36.8
Master or higher	0.29 (0.25 to 0.33)	0.45 (0.38 to 0.53)	4.72 (4.18 to 5.25)	−51.6
Steady male partner				
No/it’s complicated	1.00	1.00	7.32 (7.07 to 7.57)	0.0
Yes	0.59 (0.54 to 0.64)	0.72 (0.66 to 0.80)	5.52 (5.11 to 5.94)	−24.6
Financial coping				
Living really comfortably	1.00	1.00	5.90 (5.34 to 6.47)	0.0
Living comfortably	0.89 (0.78 to 1.01)	0.94 (0.82 to 1.07)	5.59 (5.21 to 5.98)	−5.3
Not comfortable/struggling	1.32 (1.18 to 1.48)	1.23 (1.09 to 1.38)	7.10 (6.76 to 7.43)	+20.3
Struggling	1.65 (1.45 to 1.87)	1.50 (1.31 to 1.72)	8.44 (7.81 to 9.06)	+43.1
Really struggling	2.16 (1.84 to 2.53)	1.88 (1.59 to 2.23)	10.22 (9.10 to 11.35)	+73.2
Sex with women				
Never	1.00	1.00	6.27 (6.01 to 6.52)	0.0
Yes, previous 12 months	1.80 (1.63 to 1.99)	1.90 (1.70 to 2.11)	10.86 (9.99 to 11.73)	+73.2
Yes, more 12 months ago	0.94 (0.87 to 1.02)	1.16 (1.06 to 1.26)	7.17 (6.73 to 7.62)	+14.4
Sex safe as planned				
Yes	1.00	1.00	6.57 (6.31 to 6.83)	0.0
No	1.36 (1.22 to 1.46)	1.17 (1.09 to 1.27)	7.58 (7.18 to 7.98)	+15.4
Easy to say ‘No’ to sex				
Yes	1.00	1.00	6.68 (6.43 to 6.93)	0.0
No	1.37 (1.27 to 1.47)	1.14 (1.05 to 1.24)	7.54 (7.09 to 7.99)	+12.9
Age at first sex with a man				
Don’t know	2.09 (1.57 to 2.80)	1.89 (1.40 to 2.56)	7.99 (5.94 to 10.05)	+76.8
≤13 years	2.61 (2.38 to 2.86)	2.21 (2.00 to 2.44)	9.20 (8.69 to 9.72)	+103.5
14–17 years	2.33 (2.14 to 2.64)	1.79 (1.64 to 1.96)	7.67 (7.31 to 8.02)	+69.7
≥18 years	1.00	1.00	4.52 (4.21 to 4.82)	0.0
Alcohol dependency				
No	1.00	1.00	6.72 (6.48 to 6.97)	0.0
Yes	1.44 (1.33 to 1.55)	1.14 (1.05 to 1.24)	7.49 (7.03 to 7.95)	+11.5
Stimulant use 12 months*				
No	1.00	1.00	6.21 (5.97 to 6.44)	0.0
Yes	2.09 (1.93 to 2.26)	1.82 (1.63 to 2.03)	10.54 (9.71 to 11.37)	+69.7
Heroin use 12 months				
No	1.00	1.00	6.89 (6.67 to 7.10)	0.0
Yes	3.21 (2.32 to 4.43)	1.59 (1.12 to 2.27)	10.31 (7.30 to 13.32)	+49.6
Ketamine use 12 months				
No	1.00	1.00	6.77 (6.56 to 6.99)	0.0
Yes	2.26 (1.96 to 2.62)	1.64 (1.35 to 1.98)	10.38 (8.79 to 11.97)	+53.3
Cannabis use 12 months				
No	1.00	1.00	6.91 (6.62 to 7.21)	0.0
Yes	1.40 (1.31 to 1.50)	0.98 (0.90 to 1.07)	6.91 (6.50 to 7.32)	0.0
GHB/GBL use 12 months†				
No	1.00	1.0	6.85 (6.64 to 7.07)	0.0
Yes	2.04 (1.68 to 2.47)	1.42 (1.12 to 1.79)	9.32 (7.51 to 11.12)	+36.1

* Stimulant substances include cocaine, ecstasy, amphetamines, methamphetamine, mephedrone, synthetic stimulants other than mephedrone.

† Gamma hydroxybutyrate (GHB) and gamma butyrolactone (GBL).

LAMIS, Latin American MSM Internet Survey.

Our multivariable analysis showed that, after adjusting for confounding factors (age, education, country of residence, country of birth, steady male partner status, financial coping, potential alcohol dependency, substance use, sex with women, sexual agency and age at first sex with a man), several variables were identified as factors associated with selling sex in the past year (table 2).

The odds of selling sex declined by increasing age also after adjusting for confounding: compared with those aged 18–24 years, men aged 25–29 had an estimated 24.0% lower odds (AOR 0.76, 95% CI 0.69 to 0.83) of selling sex, declining to AOR 0.17, 95% CI 0.10 to 0.30) of selling for MSM aged 55+.

The country of residency was associated with selling sex. Using Brazil as the reference category (where 4.9% had sold sex in the last 12 months) participants from almost all other countries but Uruguay had higher odds of selling sex. The country with the lowest AOR was Venezuela (AOR 1.12, 95% CI 0.89 to 1.42), while the highest was observed in Bolivia (AOR 2.66, 95% CI 2.01 to 3.51) (table 2).

Being born abroad was a characteristic positively associated with selling sex. However, the odds of selling sex at least once in the last 12 months were similar across those born abroad in Venezuela, in other LAMIS countries or elsewhere (AOR 1.48 for all categories, table 2). Financial hardship was associated with selling sex. Compared with those living really comfortably, men who reported really struggling financially had 88% higher odds of selling sex at least once in the last 12 months (AOR 1.88, 95% CI 1.59 to 2.23).

Apart from higher income, higher educational level was also associated with lower odds of selling sex. Having a master’s degree or higher incurred 55% lower odds of selling sex in the past year compared with those with only high school education or less: AOR 0.45, 95% CI 0.38 to 0.53 (table 2). Relationship status also mattered. Those with a current steady male partner had lower odds of selling sex the past year (AOR 0.72, 95% CI 0.66 to 0.80) than those who did not.

Interestingly, those who had had sex with a woman in the previous 12 months or at any point in their life also had higher odds of selling sex at least once in the previous 12 months (AOR 1.90, 95% CI 1.70 to 2.11 and AOR 1.16, 95% CI 1.06 to 1.26, respectively) (table 2). Compared with those who had their first sex with a man at 18 years or older, those who had sex at 13 years or younger or between 14 and 17 years had 121% (AOR 2.21, 95% CI 2.00 to 2.44) and 79% (AOR 1.79, 95% CI 1.64 to 1.96) higher odds of selling sex at least once in the previous 12 months, respectively (table 2). Finally, those who did not agree with the statements *I find it easy to say 'no’ to the sex I don’t want* and *The sex I have is always as safe as I want it to be* had 14% (AOR 1.14, 95% CI 1.05 to 1.24) and 17% (AOR 1.17, 95% CI 1.09 to 1.27) higher odds of selling sex at least once in the previous 12 months, respectively (table 2).

Substance and alcohol use were positively associated with selling sex. Participants classified as potentially alcohol dependent were 14% (AOR 1.14, 95% CI 1.05 to 1.24) more likely to have sold sex (table 2). Using stimulant substances was associated with 82% higher odds (AOR 1.82, 95% CI 1.63 to 2.03), GHB/GBL by 42% (AOR 1.42, 95% CI 1.12 to 1.79), ketamine use with 64% (AOR 1.64, 1.35 to 1.98) and heroin use the last 12 months with 59% higher odds (AOR 1.59, 95% CI 1.12 to 2.27). There was no significant association between cannabis use and selling sex (table 2, p>0.05). No multiplicative interaction was found between substance use, potential alcohol dependency and age (p>0.05).

Table 2 shows the relative changes in predicted probabilities of selling sex in the previous 12 months. The largest increases were observed among men initiating sex with another man at ≤13 years (103.5%), those reporting sex with women in the past 12 months (73.2%) and those reporting severe financial struggles (73.2%). Substance use was also associated with higher probabilities, ranging from a 69.7% increase among stimulant users to 36.1% among GHB/GBL users. Compared with Brazil, the highest country-level increases were observed in Bolivia (137%) and Mexico (110.6%). Predicted probabilities declined progressively with increasing age and educational attainment.

## Discussion

In this large-scale survey, including more than 53 000 MSM across 18 Latin American countries, we found that 6.9% of the respondents had sold sex in the past 12 months (10.3% among those aged 18–24) but with large variations between countries. Younger age, early sexual debut with a male partner, stimulant or depressant/dissociative substance use in the previous 12 months, low financial coping, alcohol dependency, low sexual agency, being a migrant, having had sex with a woman and country of birth were variables associated with higher odds of selling sex to men. A high educational level and having a steady male partner were associated with lower odds of selling sex.

The prevalence rates in our study are similar to those found in other web-based multicountry studies using convenience sampling in Latin America and Europe,^4 5^ but lower than in studies using response-driven sampling in Latin America.^6 7^ One possible explanation is that the latter studies were conducted in specific populations, such as among pre-exposure prophylaxis users^6^ or among those at increased risk of engaging in sex work.^7^

The prevalence of MSM selling sex in the past year differed significantly between countries, with the highest rates observed in Bolivia and the lowest in Uruguay. However, given that our study used convenience sampling, these differences may reflect variations in recruitment patterns across countries, cultural differences with regards to openness to homosexuality or unmeasured country-specific factors, such as levels of inequality, access to the internet, poverty and the legal landscape regulating (or not regulating) transactional sex as shown elsewhere.^20^ Future studies assessing sex work among MSM in Latin America should consider incorporating country-level variables into their surveys.

As corroborated by previous research,^1 3 21 22^ poor financial coping, being born abroad and having low education were significantly associated with selling sex, highlighting the intersection between selling sex and social factors. In our study, MSM, from Venezuela, were the most common group of those living in a country and born abroad, representing 42% of all MSM born abroad who sold sex. The ongoing political and economic crisis in Venezuela has forced many to leave the country under perilous conditions,^23^ increasing their economic vulnerability in the host country. Our results also indicate that, even after adjusting for other sociodemographic variables, age remained significantly associated with selling sex. Younger men were more likely to engage in sex work, a pattern that may reflect social norms favouring transactional relationships between younger and older MSM, as described by Baral *et al*.^1^

Using GHB/GBL, any stimulant substance, ketamine or heroin during the previous 12 months was associated with higher odds of selling sex, mirroring previous research.^3^,^57 18 22^ There are different possible explanations for this. A meta-synthesis of qualitative studies found that one of the reasons MSM engaged in sex work to support substance use.^24^ MSM in general and MSM who sell sex in particular also experience high levels of stigma and discrimination,^1^ and substance use can serve as a coping mechanism to deal with these challenges.^24^ Stimulant substance use among MSM has been reported as a way to enhance sexual experiences, prolong encounters and increase sexual desire.^25^ Higher levels of use among MSM engaged in sex work suggest that substances may help facilitate endurance or desire when required for commercial sexual activity. Exclusive cannabis use was not significantly associated with selling sex, possibly because it is more common and less frequently used in chemsex practices.

In addition, potential alcohol dependency was positively associated with selling sex, which is similar to results from studies conducted in LatAm and elsewhere.^1 3 5^ The fact that as much as 21% in this self-reported survey could be potentially classified as alcohol dependent, corresponds with a European multicountry study (EMIS-2017) reporting a 18.3% potential alcohol dependency prevalence.^26^ This is a concern, as alcohol addiction and substance use are risk factors for many adverse health and social outcomes, especially among young people^27 28^ and male sex workers.^1^ Another study using data from LAMIS-2018 found that multidrug use and alcohol dependency were among the syndemic conditions significantly mediating the association between sex work and condomless anal sex^29^ known to increase the risk of contracting HIV.^1^

Our results showed that 22% of the participants had their first sexual experience with a man when they were aged 13 or younger. This is an important finding, as an early sexual debut has also been identified as a predictor of living with HIV, engaging in recent condomless anal sex and having a higher number of male sexual partners among MSM populations.^30^ In addition, our data showed that an early sexual debut with a man increased the participants’ odds of selling sex, which is in line with studies conducted among young men in Sweden,^31^ MSM in Kazakhstan^18^ and MSM in 12 East and Southeast Asian countries.^17^ Since our model adjusts for many of the factors that could explain age differences in selling sex (such as substance use, educational level and financial coping), a potential explanation for this finding is exposure to childhood sexual abuse. Although not measured in LAMIS-2018, previous studies among MSM in Latin America have reported sexual abuse in childhood to range from 20% to 59% and it has been associated with selling sex later in life,^5 9 32^ also corroborated by research from Kazakhstan^18^ and India.^33^

Participants with low sexual agency also had higher odds of selling sex. About one-third, 37% and 30%, respectively, disagreed with the statements, *The sex I have is always as safe as I want it to be* and *I find it easy to say ‘no’ to sex I don’t want,* which is similar to findings from a recent study among MSM living in England.^16^ Having low sexual agency impairs people’s ability to achieve good sexual health through voluntary, safe and pleasurable sexual relationships^34^ and may be both a cause and an outcome of unsafe sexual practices.^16^ Low sexual agency among men selling sex may stem from socioeconomic vulnerabilities that restrict their ability to negotiate sexual practices.^24^ However, the cross-sectional design of this study does not allow us to establish temporality. Improving structural conditions, including legal reform of sex work, and reducing stigma towards sex work, may also play an important role. Further research is needed to clarify these influences and to codesign interventions that enhance sexual agency of men who engage in sex work, promote their safety and address their specific needs. Finally, our analysis showed that the odds of selling sex were higher for those who had sex with women. It is difficult to know whether this is due to cultural norms, societal homophobia or socio-economic factors.

### Strengths and limitations

To the best of our knowledge, this is the largest sample of MSM in Latin America. Internet-based surveys are valuable because of their potentially very high yield at very low cost. Another strength is that the survey provided a comprehensive assessment of substance use in this population. This study has limitations. The data were collected through an online survey, primarily advertised through dating apps. Thus, it is likely that MSM who do not use dating apps or even lack internet access were less likely to participate in the survey. Our study sample had higher educational levels, and since we found that people with lower levels of education were more likely to sell sex, the prevalence of selling sex may be underestimated in our data. Emerging online platforms (eg, OnlyFans, and in LGBTQ+communities, JustForFans) have contributed to the rise of remote sex work, defined here as providing sexual services virtually without physical contact (eg, via video calls or subscription-based content).^35^ This shift has important implications for how sex work is practised and understood in Latin America. Future studies should refine measures of sex work to clearly differentiate these forms and examine how online platforms influence prevalence, motivations and experiences.

The cross-sectional nature of our data does not allow us to assess causal relationships, only associations. The generalisability of our findings is also limited due to the sampling method used. However, it is important to note that there is no established sampling frame for large representative samples of MSM in Latin America or elsewhere. Another limitation is that we used overall substance use instead of substance use associated with intercourse.

## Conclusions

This large survey identifies key individual and social factors associated with selling sex among MSM in 18 Latin American countries. Social and economic vulnerabilities were associated with odds of selling sex, corroborating previous research. A fairly large proportion also reported problematic alcohol and substance use. Harm reduction and preventive interventions may be particularly needed among younger MSM. Codeveloping these interventions with the MSM community can ensure sustainability, relevance and strengthen providers’ ability to offer individualised, respectful care. Longitudinal and qualitative studies are needed to monitor long-term health and tailor interventions to individual needs.

## Supplementary material

10.1136/bmjgh-2025-021058 online supplemental file 1

10.1136/bmjgh-2025-021058 online supplemental file 2

## Data Availability

Data are available upon reasonable request.
