# Supplementary material for: Prevalence and associated factors of selling sex among men who have sex with men (MSM) in Latin America: results from the Latin American MSM Internet Survey in 18 countries (LAMIS-2018)
Source: BMJ Glob Health. 2025 Dec 19;10(12):e021058. doi: 10.1136/bmjgh-2025-021058 (PMC12718584; doi:10.1136/bmjgh-2025-021058)
Supplement: online supplemental file 1 [file bmjgh-10-12-s001.docx]

**Supplementary file 1**. Multinomial regression. Factors associated with selling sex at least once in the previous 12 months, adjusted relative risk ratios (ARRR), and 95% CI shown; reference category no selling sexing in the last twelve months, n= 51,530.

|  | 1-2 times | 3 or more |
| --- | --- | --- |
| **Characteristics** | ARRR (95% CI) | ARRR(95% CI) |
| **Age (years)** |  |  |
| 18–24 | 1.00 | 1.00 |
| 25–29 | 0.73 (0.66-0.82) | 0.82 (0.71-0.93) |
| 30–34 | 0.58 (0.50-0.67) | 0.66 (0.56-0.79) |
| 35–39 | 0.52 (0.43-0.67) | 0.50 (0.39-0.63) |
| 40–44 | 0.38 (0.29-0.50) | 0.53 (0.39-0.71) |
| 45–49 | 0.27 (0.18-0.40) | 0.27 (0.16-0.44) |
| 50–54 | 0.21 (0.12-0.36) | 0.26 (0.14-0.48) |
| 55 or more | 0.19 (0.10-0.36) | 0.16 (0.06-0.40) |
| **Country of residence** |  |  |
| Brazil | 1.00 | 1.00 |
| Argentina | 1.54 (1.28-1.86) | 1.44 (1.15-1.79) |
| Chile | 1.20 (0.97-1.49) | 1.07 (0.83-1.39) |
| Colombia | 2.27 (1.95-2.65) | 1.96 (1.63-2.35) |
| Ecuador | 2.08 (1.57-2.77) | 1.16 (0.76-1.74) |
| Mexico | 2.38 (2.07-2.74) | 2.20 (1.87-2.60) |
| Peru | 2.17 (1.69-2.80) | 1.69 (1.22-2.34) |
| Venezuela | 1.18 (0.88-1.59) | 1.07 (0.74-1.55) |
| Bolivia | 2.37 (1.66-3.45) | 3.06 (2.07-4.54) |
| Costa Rica | 1.57 (1.09-2.28) | 0.61 (0.32-1.17) |
| El Salvador | 1.81 (1.15-2.87) | 1.79 (1.04-3.07) |
| Guatemala | 1.96 (1.42-2.73) | 1.81 (1.21-2.69) |
| Honduras | 2.49 (1.72-3.59) | 1.44 (0.84-2.46) |
| Nicaragua | 2.27 (1.48-3.47) | 2.20 (1.32-3.67) |
| Panama | 1.93 (1.27-2.92) | 1.71 (1.03-2.82) |
| Paraguay | 1.92 (1.24-2.99) | 1.68 (0.96-2.92) |
| Suriname | 1.28 (0.46-3.54) | 1.54 (0.55-4.32) |
| Uruguay | 0.96 (0.56-1.63) | 0.96 (0.53-1.75) |
| **Country of birth** |  |  |
| Born in country of residency | 1.00 | 1.00 |
| Born abroad (in Venezuela) | 1.05 (0.76-1.46) | 2.22 (1.64-3.01) |
| Born abroad (other LAMIS countries) | 1.48 (1.10-1.98) | 1.48 (1.04-2.10) |
| Born abroad (other non-LAMIS countries) | 1.28 (0.78-2.11) | 1.85 (1.10-3.13) |
| **Education** |  |  |
| High school or less | 1.00 | 1.00 |
| Technical education | 0.86 (0.74-1.00) | 0.77 (0.64-0.91) |
| University | 0.67 (0.58-0.78) | 0.48 (0.41-0.57) |
| Master or higher | 0.50 (0.40-0.61) | 0.36 (0.29-0.47) |
| **Current steady male partner** |  |  |
| No/it’s complicated | 1.00 | 1.00 |
| Yes | 0.68 (0.60-0.76) | 0.80 (0.69-0.92) |
| **Financial coping** |  |  |
| Living really comfortably | 1.00 | 1.00 |
| Living comfortably | 0.98 (0.83-1.16) | 0.88 (0.72-1.07) |
| Neither comfortable nor struggling | 1.32 (1.13-1.53) | 1.01 (1.92-1.31) |
| Struggling | 1.56 (1.31-1.86) | 1.40 (1.14-1.72) |
| Really struggling | 1.72 (1.38-2.15) | 2.07 (1.62-2.63) |
| **Sex with women** |  |  |
| Never | 1.00 | 1.00 |
| Yes, previous twelve months | 1.59 (1.38-1.83) | 2.34 (2.01-2.72) |
| Yes, more than twelve months ago | 1.20 (1.08-1.34) | 1.09 (0.96-1.25) |
| **Sex always as safe as planned** |  |  |
| Yes | 1.00 | 1.00 |
| No | 1.25 (1.13-1.37) | 1.06 (0.94-1.20) |
| **Easy to say ‘No’ to unwanted sex** |  |  |
| Yes | 1.00 | 1.00 |
| No | 1.09 (0.97-1.19) | 1.25 (1.11-1.42) |
| **Age at first sex with a man** |  |  |
| Don´t know | 1.63 (1.09-2.40) | 2.34 (1.51-3.63) |
| <=13 years | 1.93 (1.71-2.19) | 2.69 (2.31-3.12) |
| 14-17 years | 1.70 (1.52-1.91) | 1.94 (1.68-2.24) |
| >= 18 years | 1.00 | 1.00 |
| **Potential alcohol dependency** |  |  |
| No | 1.00 | 1.00 |
| Yes | 1.10 (0.99-1.22) | 1.17 (1.03-1.32) |
| **Stimulant substance use**  **previous twelve months*** |  |  |
| No | 1.00 | 1.00 |
| yes | 1.60 (1.39-1.84) | 1.21 (1.88-2.60) |
| **Heroin use previous twelve months** |  |  |
| No | 1.00 | 1.00 |
| Yes | 1.54 (0.97-2.45) | 1.62 (1.01 – 2.58) |
| **Ketamine use previous twelve months** |  |  |
| No | 1.00 | 1.00 |
| Yes | 1.53 (1.19-1.97) | 1.75 (1.35-2.27) |
| **Cannabis use previous twelve months** |  |  |
| No | 1.00 | 1.00 |
| Yes | 1.01 (0.90-1.14) | 0.97 (0.84-1.11) |
| **GHB/GBL use previous twelve months†** |  |  |
| No | 1.00 | 1.0 |
| Yes | 1.19 (0.86-1.65) | 1.69 (1.24-2.30) |

*Stimulant substances include cocaine, ecstasy, amphetamines, methamphetamine, mephedrone, synthetic stimulants other than mephedrone. †Gamma hydroxybutyrate (GHB) and gamma butyrolactone (GBL).
