# Supplementary material for: Prevalence and associated factors of selling sex among men who have sex with men (MSM) in Latin America: results from the Latin American MSM Internet Survey in 18 countries (LAMIS-2018)
Source: BMJ Glob Health. 2025 Dec 19;10(12):e021058. doi: 10.1136/bmjgh-2025-021058 (PMC12718584; doi:10.1136/bmjgh-2025-021058)
Supplement: online supplemental file 2 [file bmjgh-10-12-s002.docx]

**Supplementary file 2**. Comparison between those included and excluded from the analysis.

| **Characteristics** | **Included** | | **Excluded** | |
| --- | --- | --- | --- | --- |
|  | n | % | n | % |
| **Age (years)*** |  |  |  |  |
| 18–24 | 17,864 | 33.5 | 3,189 | 33.5 |
| 25–29 | 13,314 | 25.0 | 2,235 | 23.5 |
| 30–34 | 8,666 | 16.2 | 1,548 | 16.3 |
| 35–39 | 5,465 | 10.2 | 1,023 | 10.7 |
| 40–44 | 3,209 | 6.0 | 571 | 6.0 |
| 45–49 | 2,152 | 4.0 | 458 | 4.8 |
| 50–54 | 1,472 | 2.8 | 281 | 3.0 |
| 55 or more | 1,200 | 2.2 | 213 | 2.2 |
| Total | 53,342 | 100.00 | 9,518 | 100.00 |
| **Education*** |  |  |  |  |
| High school or less | 5,636 | 10.6 | 1,174 | 12.4 |
| Technical education | 11,342 | 21.3 | 2,295 | 24.2 |
| University | 26,570 | 49.9 | 4,416 | 46.6 |
| Master or higher | 9,693 | 18.2 | 1,599 | 16.9 |
| Total | 53,241 | 100.00 | 9,484 | 100.00 |
| **Country of residency*** |  |  |  |  |
| Brazil | 15,113 | 28.3 | 2,619 | 27.5 |
| Argentina | 4,575 | 8.6 | 787 | 8.3 |
| Chile | 4,211 | 7.9 | 618 | 6.5 |
| Colombia | 6,680 | 12.5 | 1,301 | 13.7 |
| Ecuador | 1,168 | 2.2 | 225 | 2.4 |
| Mexico | 12,227 | 22.9 | 2,280 | 24.0 |
| Peru | 1,591 | 3.0 | 343 | 3.6 |
| Venezuela | 2,031 | 3.8 | 337 | 3.5 |
| Bolivia | 591 | 1.1 | 124 | 1.3 |
| Costa Rica | 869 | 1.6 | 119 | 1.3 |
| El Salvador | 465 | 0.9 | 89 | 0.9 |
| Guatemala | 929 | 1.7 | 193 | 2.0 |
| Honduras | 536 | 1.0 | 84 | 0.9 |
| Nicaragua | 435 | 0.8 | 73 | 0.8 |
| Panama | 614 | 1.2 | 124 | 1.3 |
| Paraguay | 472 | 0.9 | 91 | 1.0 |
| Suriname | 170 | 0.3 | 31 | 0.3 |
| Uruguay | 665 | 1.2 | 80 | 0.8 |
| Total | 53,342 | 100.00 | 9,518 | 100.00 |
| **Country of birth*** |  |  |  |  |
| Born in country of residence | 50,912 | 95.6 | 9,023 | 94.9 |
| Born abroad (in Venezuela) | 988 | 1.9 | 209 | 2.3 |
| Born abroad (other LAMIS countries) | 971 | 1.8 | 212 | 2.2 |
| Born abroad (non-LAMIS countries) | 414 | 0.8 | 55 | 0.6 |
| Total | 53,285 | 100.00 | 9,499 | 100.00 |
| **Current steady male partner** |  |  |  |  |
| Yes | 13,901 | 26.1 | 2,503 | 26.4 |
| No/It’s complicated | 39,335 | 73.9 | 6,971 | 73.6 |
| Total | 53,236 | 100.00 | 9,474 | 100.00 |
| **Financial coping*** |  |  |  |  |
| Living really comfortably | 7,256 | 13.6 | 1,664 | 17.6 |
| Living comfortably | 14,869 | 28.0 | 2,537 | 26.8 |
| Neither comfortable nor struggling | 21,425 | 40.3 | 3,650 | 38.5 |
| Struggling | 7,100 | 13.4 | 1,188 | 12.5 |
| Really struggling | 2,515 | 4.7 | 438 | 4.6 |
| Total | 53,165 | 100.00 | 9,477 | 100.00 |
| **Sex with women*** |  |  |  |  |
| Never | 33,214 | 62.6 | 5,613 | 59.4 |
| Yes, in the previous twelve months | 4,660 | 8.8 | 1,094 | 11.6 |
| Yes, more than twelve months ago | 15,182 | 28.6 | 2,735 | 29.0 |
| Total | 53,056 | 100.00 | 9,442 | 100.00 |
| **Sex always as safe as planned*** |  |  |  |  |
| Yes | 36,865 | 69.2 | 6,394 | 67.3 |
| No | 16,433 | 30.8 | 3,113 | 32.7 |
| Total | 53,298 | 100.00 | 9,507 | 100.00 |
| **Easy to say ‘No’ to unwanted sex*** |  |  |  |  |
| Yes | 40,379 | 75.9 | 7,114 | 74.9 |
| No | 12,855 | 24.1 | 2,387 | 25.1 |
| Total | 53,234 | 100.00 | 9,501 | 100.00 |
| **Potential alcohol dependency*** |  |  |  |  |
| No | 41,780 | 78.9 | 7,288 | 77.5 |
| Yes | 11,152 | 21.1 | 2,112 | 22.5 |
| Total | 52,932 | 100.00 | 9,400 | 100.00 |
| **Age at first sex with a man*** |  |  |  |  |
| Don’t know | 663 | 1.2 | 98 | 1.0 |
| <=13 years | 11,784 | 22.2 | 1,957 | 20.7 |
| 14–17 years | 19,132 | 36.1 | 3,489 | 36.9 |
| >= 18 years | 21,472 | 40.5 | 3,921 | 41.4 |
| Total | 53,051 | 100.00 | 9,465 | 100.00 |
| **Stimulant substance use** |  |  |  |  |
| **previous twelve months** |  |  |  |  |
| No | 45,193 | 85.3 | 8,055 | 85.4 |
| Yes | 7,819 | 14.7 | 1,382 | 14.6 |
| Total | 53,012 | 100.00 | 9,437 | 100.00 |
| **Heroin use previous twelve months** |  |  |  |  |
| No | 52,728 | 99.5 | 9,371 | 99.4 |
| Yes | 241 | 0.5 | 56 | 0.6 |
| Total | 52,969 | 100.00 | 9,427 | 100.00 |
| **Ketamine use** **previous twelve months*** |  |  |  |  |
| No | 51,407 | 97.0 | 9,158 | 97.2 |
| Yes | 1,568 | 3.0 | 267 | 2.8 |
| Total | 52,969 | 100.00 | 9,431 | 100.00 |
| **Cannabis use previous twelve months*** |  |  |  |  |
| No | 36,773 | 69.4 | 6,902 | 73.1 |
| Yes | 16,231 | 30.6 | 2,534 | 26.9 |
| Total | 53,004 | 100.00 | 9,436 | 100.00 |
| **GHB/GBL use previous twelve months** |  |  |  |  |
| No | 51,990 | 98.2 | 9,253 | 98.2 |
| Yes | 958 | 1.8 | 173 | 1.8 |
| Total | 52,948 | 100.00 | 9,426 | 100.00 |

*Chi 2, p<0.05.
